# Denticleless E3 Ubiquitin Protein Ligase Homolog as a Potential Biomarker for Adrenocortical Carcinoma Screening

**DOI:** 10.2174/0118715303334496250518033852

**Published:** 2025-06-20

**Authors:** Xin Yan, Li-Xing Pang, Xiao Lu, Sheng Chen, Li Li, Xing-Huan Liang, De-Cheng Lu, Zuo-Jie Luo

**Affiliations:** 1 Department of Endocrinology, The Second People’s Hospital of Nanning City, The Third Affiliated Hospital of Guangxi Medical University, Nanning 530031, Guangxi Province, China;; 2 Department of Endocrinology, The First Affiliated Hospital of Guangxi Medical University, Nanning 530021, Guangxi Province, China

**Keywords:** Adrenocortical carcinoma, bioinformatics, biomarkers, adrenocortical tumors, core genes, immunohistochemistry, survival analysis

## Abstract

**Objective:**

This study aims to investigate the potential of denticleless E3 ubiquitin protein ligase homolog (*DTL*) as a biomarker for adrenocortical carcinoma (ACC) detection through bioinformatics analysis and experimental validation.

**Methods:**

Differentially expressed genes (DEGs) between ACC and adrenocortical adenoma (ACA) were identified through bioinformatics analysis. A protein-protein interaction (PPI) network was constructed using Cytoscape software, and core genes were screened with the CytoHubba MCODE plug-in. Survival analysis was performed using the University of ALabama at Birmingham CANcer (UALCAN) data analysis portal. Immunohistochemistry was employed to assess DTL expression in adjacent normal tissues, ACA, and ACC.

**Results:**

Two gene expression series (GSEs) retrieved from the Gene Expression Omnibus (GEO) database yielded 115 DEGs. Using the PPI network, three core genes were identified, among which *DTL* and *TPX2* were highly expressed in ACC. Notably, *DTL* had the highest core gene score. Elevated *DTL* expression in individuals with ACC was significantly associated with a poor prognosis (*P* < 0.0001). Immunohistochemistry analysis revealed a significantly higher positive expression rate and a strong positive expression rate of *DTL* in ACC compared to ACA (χ2 = 11.708, *P* < 0.01). The positive expression rate of *DTL* in both ACC and ACA was significantly higher than in the adjacent normal adrenal cortex (*P* < 0.01). The expression of DTL followed a gradient, being highest in ACC, followed by ACA, and lowest in the normal adrenal cortex adjacent to the tumor. Additionally, *DTL* protein expression was significantly correlated with tumor size and infiltration metastasis (*P* < 0.05). Individuals with high *DTL* expression had significantly shorter survival times than those with low *DTL* expression (*P* < 0.05).

**Discussion:**

The CRL4CDT2 ubiquitin ligase complex, also known as DCX (DTL), is related to DNA synthesis. The findings suggest that DTL may serve as a key oncogene in multiple malignancies, including ACC and GC, making it a potential therapeutic target for cancer treatment. The findings collectively indicate that DTL is frequently overexpressed in various malignancies, highlighting its potential role in tumorigenesis. The findings of our study are consistent with these observations. The precise mechanisms by which Cdt2 overexpression drives tumorigenesis are not fully understood. However, one proposed mechanism involves the ability of the CRL4Cdt2 complex to promote cell cycle progression by reducing the basal steady-state levels of the cell cycle inhibitor p21, thereby impairing p53-mediated DNA damage repair mechanisms. In addition, DTL has been shown to play a key role in promoting tumor cell proliferation, migration, and invasion through both p53-dependent and p53-independent pathways.

**Conclusion:**

*DTL* exhibits potential as a novel biomarker for distinguishing between benign and malignant adrenocortical tumors and may serve as a prognostic indicator for ACC.

## 1 INTRODUCTION

Adrenocortical carcinoma (ACC) is a rare but aggressive malignancy associated with significant morbidity in patients with adrenocortical tumors, with an estimated incidence of approximately 0.7–2 per million individuals annually [1]. Advances in imaging modalities, such as cross-sectional imaging techniques, have facilitated the increased detection of adrenal masses during evaluations for non-adrenal conditions, commonly referred to as adrenal incidentalomas [2]. Although the majority of adrenal incidentalomas are benign and non-functional, they pose a risk of malignant transformation and endocrine dysfunction. ACC accounts for approximately 15% of adrenal incidentalomas [3].

At present, there is no universally accepted standard for the differential diagnosis of adrenal incidentalomas. The Weiss system remains the most commonly used histopathological gold standard for evaluating ACT malignancy [4]. However, its limitations—including interobserver variability, reduced sensitivity in pediatric populations, and diagnostic uncertainty in tumors with intermediate Weiss scores (*e.g.*, scores of 3-4)—are well-documented [5]. A multicenter study by Riedmeier *et al*. revealed that a considerable proportion of pediatric ACC cases were misclassified using the Weiss criteria, leading to delayed clinical interventions [4]. These challenges have driven efforts to identify molecular markers that can enhance diagnostic accuracy. Recent genomic profiling studies have identified recurrent mutations in genes such as *TP53*, *CTNNB1*, and *ZNRF3* in ACC; however, their clinical utility as independent, standalone markers remains constrained by heterogeneity and the lack of standardized assays.

In this context, the exploration of cell cycle regulators as biomarkers offers a promising avenue for improving ACC diagnosis. Denticleless E3 ubiquitin protein ligase homolog (*DTL*), also known as *CDT2* or *DCAF2*, is a critical component of the CRL4Cdt2 ubiquitin ligase complex [6-8]. This complex orchestrates the degradation of key cell cycle regulators, including p21, SET8, and Cdt1, thereby ensuring proper DNA replication and damage repair [9]. By ensuring the timely proteolysis of these substrates, *DTL* maintains genomic stability and prevents replication stress, a mechanism frequently exploited in tumorigenesis [10]. Emerging evidence has linked *DTL* dysregulation to oncogenesis across diverse malignancies. For example, Chen *et al*. noted that *DTL* silencing in hepatocellular carcinoma (HCC) induces G2/M phase arrest and suppresses tumor growth through p21 stabilization, highlighting its potential role as a therapeutic target [7]. Despite these insights, the functional significance of DTL in adrenocortical tumors remains unexplored, representing a critical gap in both basic and translational research.

To address this knowledge gap, the present study employed an integrative approach combining bioinformatics analysis of public gene expression datasets and immunohistochemical validation in clinical specimens. *DTL* was identified as a key ACC-associated gene through bioinformatics screening, and the expression of *DTL* in benign and malignant adrenocortical tumors was subsequently verified using immunohistochemistry. The relationship between *DTL* expression and clinicopathological features of adrenocortical tumors, including tumor proliferation activity, was analyzed to explore the role of *DTL* in the occurrence and development of human ACC and to provide a theoretical basis for improving the diagnostic accuracy and treatment strategies for ACC.

## 2 MATERIALS AND METHODS

### 2.1 Gene Expression Profile Data

Two mRNA expression datasets, GSE12368 and GSE33371, for adult ACC were retrieved and downloaded from the Gene Expression Omnibus (GEO) database (https://www.ncbi.nlm.nih.gov/geo/). All mRNA expression profiles were generated using the GPL570 platform (Affymetrix Human Genome U113 Plus 2.0 Array). The GSE12368 dataset consisted of 12 ACC samples and 16 adrenocortical adenoma (ACA) samples, while the GSE33371 dataset included 33 ACC samples and 22 ACA samples. To ensure data reliability and consistency, background signal correction and normalization preprocessing on the raw CEL files were conducted using the affy package in RStudio (Version 3.5.3). REMARK guidelines were followed.

### 2.2 Screening of Differentially Expressed Genes (DEGs)

The gene expression matrix was divided into the case group (ACC) and the control group (ACA) for the identification of differentially expressed genes (DEGs). The statistical significance of gene expression difference was calculated using the limma package in R [11], and the Benjamini-Hochberg (BH) method was used for multiple testing correction. DEGs were defined based on the following criteria: *P* < 0.05, |logFC| ≥ 2. The R software package pheatmap [7] was used to generate a heatmap to visualize the expression patterns of the DEGs.

### 2.3 Functional Annotation and Pathway Enrichment Analysis

To investigate the biological significance of the identified DEGs, the Gene Ontology (GO) [12] and the Kyoto Encyclopedia of Genes and Genomes (KEGG) [13] pathway enrichment analyses were conducted. Functional enrichment of the upregulated and downregulated DEGs was performed using the Database for Annotation, Visualization and Integrated Discovery (DAVID) [14] (Version 6.8) enrichment analysis tool (https://david-d.ncifcrf.gov/). This analysis enabled the identification of key biological processes, molecular functions, cellular components, and signaling pathways associated with the dysregulated genes in ACC.

### 2.4 Protein-Protein Interaction (PPI) Network and Module Selection of DEGs

The Search Tool for Retrieval of Interacting Genes/Proteins (STRING) database [15] (Version: 10.0) (http://www.string-db.org/) was utilized to investigate potential interactions among the proteins encoded by the DEGs. The PPI network model of DEGs was constructed using Cytoscape (Version 3.6.0) [16]. To identify key functional modules in the PPI network, the MCODE plug-in, which detects highly interconnected gene clusters, was used. Additionally, the CytoHubba plug-in was utilized to identify core genes. The top 30 hub genes were selected using four topological ranking methods: radiality, betweenness, stress, and clustering coefficient. The common core genes were identified and ranked using Venn diagram analysis (http://bioinformatics.psb.ugent.be/webtools/Venn/).

### 2.5 Survival Analysis of the Core Gene DTL

The University of ALabama at Birmingham CANcer (UALCAN) data analysis portal (http://ualcan.path.uab.edu/ analysis.html) was used to evaluate the prognostic significance of DTL. The survival data were obtained from The Cancer Genome Atlas (TCGA). Patients with ACC were categorized into high and low *DTL* expression groups based on the median expression level of *DTL* (threshold: 3rd quartile). Additionally, UALCAN was used to download the mRNA and clinical data from the TCGA database to evaluate the correlation between *DTL* expression and tumor grade. This analysis aimed to determine whether *DTL* could be used as a molecular marker for ACC, particularly about tumor progression and prognosis.

### 2.6 Expression of *DTL* in ACC

#### 2.6.1 Clinical Data

A total of 57 paraffin-embedded adrenocortical tumor specimens were obtained from the First Affiliated Hospital of Guangxi Medical University, China, between January 2000 and September 2021. The diagnosis had been confirmed by pathology after surgical resection and skin adrenal biopsy. Among these, 18 specimens were diagnosed as ACC based on the Weiss histopathological diagnostic criteria [4]. The other 31 cases of ACA and 8 adjacent normal adrenal tissue specimens were included as controls.

The study sample consisted of 49 patients with adrenocortical tumors (19 males and 30 females) with an average age of 39.5 ± 2.07 years and a male-to-female ratio of 1:1.6 Demographic and Clinicopathological Characteristics of ACC Cases:

The 18 cases of ACC consisted of 3 children under 10 years of age and 15 adults. The tumor diameter was > 5 cm in 16 patients and < 5 cm in 2 patients. As per the TNM stage, there was 1 patient in Stage I, 8 patients in Stage II, 2 patients in Stage III, and 7 patients in Stage IV. Regarding metastatic status, 2 patients had lymph node metastasis, while there were 10 patients without lymph node metastasis and 6 with unclear lymph node metastasis. 3 patients had distant metastasis, and 15 patients without distant metastasis. Seven patients had typical clinical manifestations of hormonal overproduction, 4 patients showed local compression symptoms, 4 patients had non-specific clinical manifestations, and 3 patients had positive findings detected during routine physical examinations.

The collected pathological specimens were subjected to immunohistochemical (IHC) staining. All histopathological sections were independently reviewed by senior pathologists to ensure diagnostic accuracy.

#### 2.6.2 Determination of Immunohistochemical Results

A double-scoring semi-quantitative integration method based on staining intensity and the proportion of positive cells was used to assess DTL expression in adrenocortical tumors. Staining Characteristics

Localization: DTL was primarily expressed in the nucleus, with secondary expression observed in the cytoplasm.

Positive Staining: Samples exhibiting clear brown or brown granular deposits were considered positive.

##### 2.6.2.1 Scoring Criteria

(A) Staining Intensity Score: The varying degrees of staining of immune-positive cells were scored as follows: no staining was scored as 0 points; mild staining was scored as 1 point; moderate staining was scored as 2 points; and intense staining was scored as 3 points.

(B) Percentage of Positive Cells: A total of 10 representative areas were randomly selected from the upper, lower, left, right, and central regions of each slide for cell counting. In each area, the proportion of DTL-positive cells in a total of 1000 cells was scored as follows: < 10% positive cells was considered 0 points; 10%-25% was scored as 1 point; 26%-50% was scored as 2 points; and > 50% was scored as 3 points.

Final Immunohistochemical Score: A semi-quantitative analysis was performed by multiplying the staining intensity (A) by the percentage of positive cells (B). A final score of 0-1 points was considered “-” (Negative); a score of 2–3 points was considered “+” (Low expression); a score of 4–6 points was considered “++” (Moderate expression); and a score of > 6 was considered “+++” (High expression). For analysis purposes, low expression was indicated by “-” or “+” while high expression was indicated by “++” or “+++”.

All immunohistochemical slides were independently reviewed by two senior pathologists in a double-blind manner. Any disagreements were resolved through discussion to arrive at a consensus in the final scoring.

#### 2.6.3 Statistical Analysis

All data were digitized and processed using Microsoft Excel for initial collation and SPSS 22.0 for Windows for statistical analysis. The significance level was set at α = 0.05. The chi-square test was used for categorical variable comparisons between groups; Fisher’s exact probability method was applied when expected frequencies in contingency tables were small (n < 5); and the rank sum test (Mann-Whitney U test or Kruskal-Wallis test) was used for non-parametric comparisons of continuous or ordinal data between groups. GraphPad Prism 7.0 was utilized to perform Kaplan-Meier survival analysis, comparing overall survival (OS) between groups stratified by DTL expression levels. Log-rank tests were used to assess statistical significance in survival differences.

## 3 RESULTS

### 3.1 DEGs

A total of 115 DEGs were identified in ACC compared to non-cancerous adrenal cortex tissues. Hierarchical clustering of 45 ACC and 38 ACA samples revealed 62 upregulated and 53 downregulated genes (Figs. **1A** and **B**).

### 3.2 Cluster Gene Screening

Using Cytoscape, a PPI network consisting of 109 nodes and 772 edges was constructed (Fig. **2A**). The most significant module within this network is shown in Fig. (**2B**).

### 3.3 Core Gene Screening

The four centrality algorithms, namely, radiality, betweenness, stress, and clustering coefficient, were applied using the CytoHubba plug-in in Cytoscape to identify the key genes. The top 30 DEGs from each method were extracted, and the four groups of DEGs were intersected using the online Venn diagram tool (http://jvenn.toulouse.inra.fr/app/example.html). The results identified three core genes: DTL, TPX2, and RAD51AP1 (Fig. **3A**). Notably, *DTL* ranked first across all four algorithms (Fig. **3B**), suggesting its pivotal role in ACC pathogenesis.

### 3.4 Effect of Core Gene *DTL* Expression on the Prognosis of Adrenocortical Tumors

Using the UALCAN database, we analyzed the correlation between mRNA expression and the overall survival (OS) rate in patients with ACC. High DTL expression was significantly associated with poor prognosis (*P* < 0.0001) (Fig. **4B**). To further validate this, mRNA and clinical data were downloaded from the TCGA database, and UALCAN was used to verify the correlation between *DTL* expression and tumor grade. The results indicated that *DTL* expression in Stage IV was significantly higher than that in Stages I, II, and III (Fig. **4A**), suggesting that *DTL* may play a role in tumor progression and metastasis, reinforcing its potential as a prognostic biomarker in ACC.

### 3.5 Expression and Clinical Significance of DTL in ACC

#### 3.5.1 DTL Expression in Adrenocortical Tumors

The DTL protein was predominantly localized in the nucleus of glandular epithelial cells, with brown-positive staining particles. There was a progressive increase in DTL expression from normal adrenal cortex tissue to ACA and ACC (Figs. **5A**-**C**).

In ACC tissues (n = 18), the positive expression rate of DTL was 88.89%, with high expression in 22.22% (4/18 cases), low expression in 66.67% (12/18 cases), and decreased or absent in 11.11% (2/18 cases).

In ACA tissues (n = 31), 38.7% (12/31 cases) were positive, but all had low expression, while the remaining 19 cases were negative. In normal adrenal cortex tissues adjacent to the tumor, there was almost no positive expression.

The positive and strong positive expression rates of DTL in ACC tissues were significantly higher than in ACA tissues (χ2 = 11.708, *P* < 0.01) (Table **1**). Additionally, the positive expression rate of DTL in ACC was significantly higher than in adjacent normal adrenal cortex tissues (*P* < 0.01). DTL positive expression rate in ACA was also significantly higher than in adjacent normal adrenal cortex tissues (*P* < 0.05) (Table **1**).

#### 3.5.2 Correlation Between DTL Expression and Clinicopathological Features of Adrenocortical Tumors

The expression of DTL protein was not significantly correlated with gender, age, or clinical stage (*P* > 0.05), but there was a significant association with tumor size, invasion, and metastasis (*P* < 0.05). Patients with a tumor diameter > 5 cm or those with invasion and metastasis showed significantly higher DTL expression (Table **2**).

#### 3.5.3 Correlation Between DTL Expression and Prognosis in ACC

A total of 18 patients with ACC were followed up for 6–89 months (median follow-up time: 39.5 months). Tumor-related mortality was 27.8% (5/18 cases). DTL protein expression levels were significantly associated with overall survival (*P* < 0.05). Patients in the high DTL expression group had a significantly shorter survival time than those in the low DTL expression group. The survival time in the high DTL expression group was (26 ± 7.2) months, while it was (76.3 ± 8.2) months in the low DTL expression group. The 3-year survival rate of patients with ACC also differed significantly, with < 30% in the high DTL expression group and > 80% in the low DTL expression group (Fig. **6**). These findings suggest that high DTL expression is associated with poorer prognosis in ACC, reinforcing its potential role as a biomarker for tumor progression and prognosis prediction.

## 4 DISCUSSION

ACC is considered a highly malignant tumor of the endocrine system, characterized by early metastasis and poor prognosis. Identifying molecular markers of ACC that can be used for early diagnosis and targeted treatment is crucial in order to enhance patient outcomes.

In this study, *DTL* was highlighted as a key gene implicated in ACC pathogenesis. The protein expression output of DTL is commonly referred to as CDT2. Since the function of free CDT2 is not yet clear, most of the current research has focused on studying the bound form of CDT2, that is, the CUL4-based E3 ubiquitin ligase CRL4CDT2 [17]. The CRL4CDT2 ubiquitin ligase complex, also known as DCX (DTL), is unique among ubiquitin ligases as its function is related to DNA synthesis [17]. It ubiquitinates key cellular substrates, leading to their subsequent proteasomal degradation during the S-phase of the cell cycle in response to DNA damage [18]. CRL4CDT2 is composed of Cullin 4 (CUL4A or CUL4B), which acts as a scaffold protein; DNA Damage Binding Protein 1 (DDB1), which functions as an adaptor; and CDT2, which serves as the substrate receptor, playing a crucial role in determining substrate specificity [10]. CDT2 is the most active and key component of the E3 ubiquitin ligase CRL4CDT2. In recent years, studies have shown that the E3 ubiquitin ligase CRL4CDT2 can regulate a variety of substrates that play a key role in the physiological cell cycle and DNA damage response [17]. Given its central role in tumor progression, CRL4CDT2-mediated DTL overexpression may contribute to ACC malignancy by promoting uncontrolled cell proliferation and genomic instability. Targeting this ubiquitin ligase pathway could serve as a potential therapeutic strategy for ACC.

*DTL*, also known as L2DTL (Lethal(2) denticleless protein homolog), is a human ortholog of the Drosophila lethal(2) denticleless gene (l(2)dt1). The gene was named based on its role in Drosophila development, where homozygous mutations result in embryonic lethality due to the absence of the ventral dentate band [19]. Bioinformatics analysis has identified that human *L2DTL* as an ortholog of Drosophila l(2)dtl, encoding the WD40 repeat protein [20], which contains a Nuclear Localization Signal (NLS), a potential PEST (proline [P], glutamic acid [E], serine [S] and threonine [T]) sequence [21], a KEN box signal, and two D-box signals. Mitotic cyclins, PTTG1/Securin, and other cell cycle regulators, which are targeted for degradation during cell cycle progression, have similar protein characteristics [22, 23]. Similar to Drosophila l(2)dt1, human *L2DTL* is highly expressed in multiple fetal tissues, indicating its involvement in embryonic development. Studies on *L2DTL* knockout mice have demonstrated that its loss leads to early embryonic lethality [24]. These findings suggest that *L2DTL* is involved in cell cycle regulation and plays an important role in cell proliferation during embryonic development.

*DTL* plays a crucial role in maintaining genomic stability, primarily through its function within the CRL4CDT2 E3 ubiquitin ligase complex. *DTL* knockdown has been shown to consistently result in significant replication stress and genomic instability [25]. Conversely, *DTL* overexpression leads to a decrease in substrate levels of its target proteins, leading to cell cycle dysregulation and contributing to pathological conditions, including tumorigenesis [26]. Studies have evidenced the importance of precise regulation of CRL4CDT2 activity, as abnormal regulation of *DTL* has been observed in a variety of tumors [27] and *DTL* has recently been identified as a target for oncogenic viruses [28]. Studies have also shown that the expression of *DTL* is increased in invasive hepatocellular carcinoma (HCC), and its levels are positively correlated with tumor grade and a high mortality rate [24] underscoring its potential as a biomarker for aggressive cancers.

In order to study DTL protein expression in adrenocortical tumors, immunohistochemical analysis on ACC, ACA, and adjacent normal tissues was performed. The result regarding DTL localization was that the protein showed brown nuclear staining in both ACC and ACA tissues, which was consistent with the findings of Pan *et al* [24]. The expression rates of DTL were 88.89% in ACC, 38.7% in ACA, and almost no detectable expression in normal adrenocortical tissues adjacent to the tumor.

The trend was that DTL expression increased progressively from normal adrenocortical tissues adjacent to the tumor to ACA to ACC, suggesting a strong association between DTL upregulation and adrenocortical tumor progression. The oncogenic role of DTL overexpression has been reported in gastric cancer (GC), For instance, Li *et al* [29] found that DTL was overexpressed in GC tissues when compared with adjacent normal tissues. Compared with normal gastric mucosal cells, seven gastric cancer cell lines overexpressed DTL. Kobayashi *et al* [30] detected DTL protein overexpression in 57% (4/7) of GC cell lines and 42% (42/100) of primary GC tumor samples (42/100 cases; 42%). DTL downregulation inhibited cell proliferation, migration, and invasion in GC cell lines, suggesting its role in tumor aggressiveness. These findings suggest that *DTL* may serve as a key oncogene in multiple malignancies, including ACC and GC, making it a potential therapeutic target for cancer treatment.

In another study, Pan *et al*. [24] reported that *L2DTL* was overexpressed in 59% of 270 cases of resected, unifocal primary liver cancer and in all four examined hepatocellular carcinoma cell lines (Hep3B, Huh7, HA22T, and SKHep-1), whereas its expression was rare (2%) in normal liver tissue. Similarly, Ueki *et al*. [31] demonstrated the upregulation of *DTL*/*RAMP* in the majority of breast cancer cases and across all breast cancer cell lines that they examined using semi-quantitative RT-PCR and Northern blot analysis. Moreover, silencing *DTL*/*RAMP* expression in T47D and HBC4 breast cancer cells *via* small interfering RNA effectively inhibited its expression and induced G2/M phase arrest, thereby inhibiting cancer cell proliferation. These findings collectively indicate that *DTL* is frequently overexpressed in various malignancies, highlighting its potential role in tumorigenesis. The findings of our study are consistent with these observations.

Additionally, in this study, there was no significant correlation between *DTL* expression and clinicopathological features, such as gender, age, or clinical stage of adrenocortical tumors. However, *DTL* expression was significantly associated with tumor size, invasion, and metastasis. Patients with ACC in the high DTL expression group had a significantly shorter survival time when compared to those in the low DTL expression group.

Kobayashi *et al*. [30] found that *DTL* overexpression in gastric cancer was significantly linked to lymph node invasion, deeper tumor infiltration, and higher recurrence rates. Similarly, Pan *et al*. [24] observed that elevated expression of *DTL* in HCC was associated with high alpha-fetoprotein (AFP) levels, larger tumor diameter (> 5 cm), and advanced clinical stage. They also found that patients with HCC and high *DTL* expression had a lower ten-year cumulative survival rate than those with HCC and low *DTL* levels. Furthermore, patients with HCC who had both high *DTL* expression and a P53 mutation had a worse prognosis. Functional studies showed that the knockdown of *DTL* significantly reduced the invasion potential of HeLa and HA22T cells [24] suggesting that *DTL* overexpression could promote tumor cell invasiveness. The findings in our present study align with these results.

The *DTL* gene is evolutionarily conserved from nematodes to humans. Under both normal and stress conditions, the CUL4-based E3 ligase (CRL4) regulates the degradation of *DTL* by targeting key substrates, including the replication licensing factor (CDT1), the cell cycle control protein (p21), and the chromatin modification factor (SET8). Through these interactions, *DTL* plays a fundamental role in the regulation of the cell cycle. The precise mechanisms by which Cdt2 overexpression drives tumorigenesis are not fully understood. However, one proposed mechanism involves the ability of the CRL4Cdt2 complex to promote cell cycle progression by reducing the basal steady-state levels of the cell cycle inhibitor p21, thereby impairing p53-mediated DNA damage repair mechanisms [32, 33]. In addition, *DTL* has been shown to play a key role in promoting tumor cell proliferation, migration, and invasion through both p53-dependent and p53-independent pathways [30].

## CONCLUSION

In conclusion, *DTL* shows promise as a novel biomarker for distinguishing between benign and malignant adrenocortical tumors and for prognostic assessment in ACC. Our findings suggest that *DTL* could contribute to tumorigenesis in ACC, playing a role in its occurrence and progression. However, this study is based on a limited sample size, necessitating further validation through larger samples and additional tissue-based polymerase chain reaction and western blot to ensure more accurate results. In future research, we plan to explore the relationship between *DTL* and ACC cells, focusing on the specific molecular and cellular mechanisms through which *DTL* promotes tumor proliferation, invasion, and metastasis. This can provide new therapeutic targets for the diagnosis, treatment, and prognosis of ACC, as well as offer new insights for developing drugs for treating this malignancy.

## Figures and Tables

**Fig. (1):**
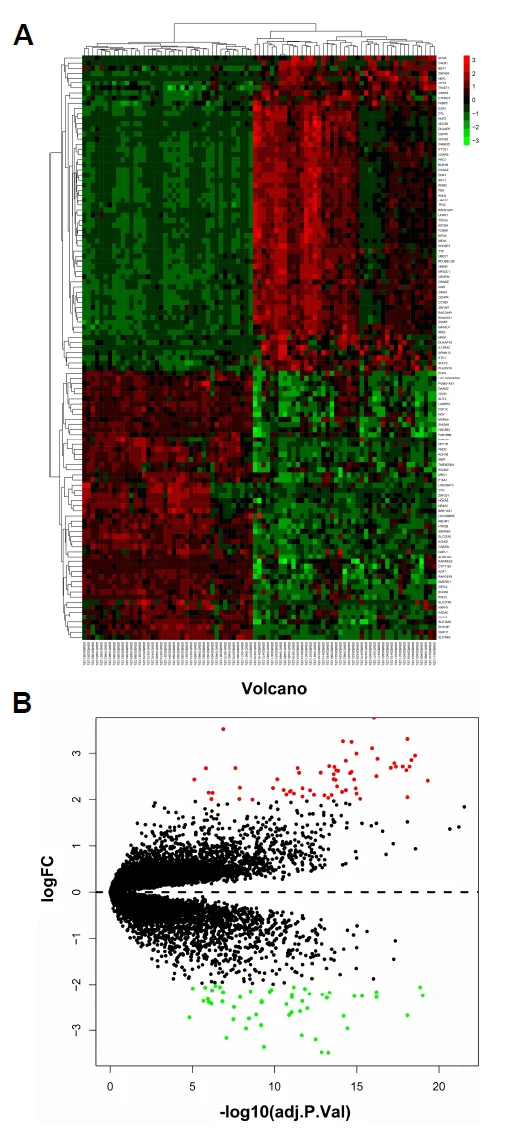
(**A**) Heatmap of DEGs between ACC (45 cases) and ACA (38 cases). The left side denotes ACA samples, while the right side denotes ACC samples. (**B**) Volcano plot of DEGs between ACC (45 cases) and ACA (38 cases). Red and green indicate upregulated and downregulated differentially expressed genes, respectively.

**Fig. (2):**
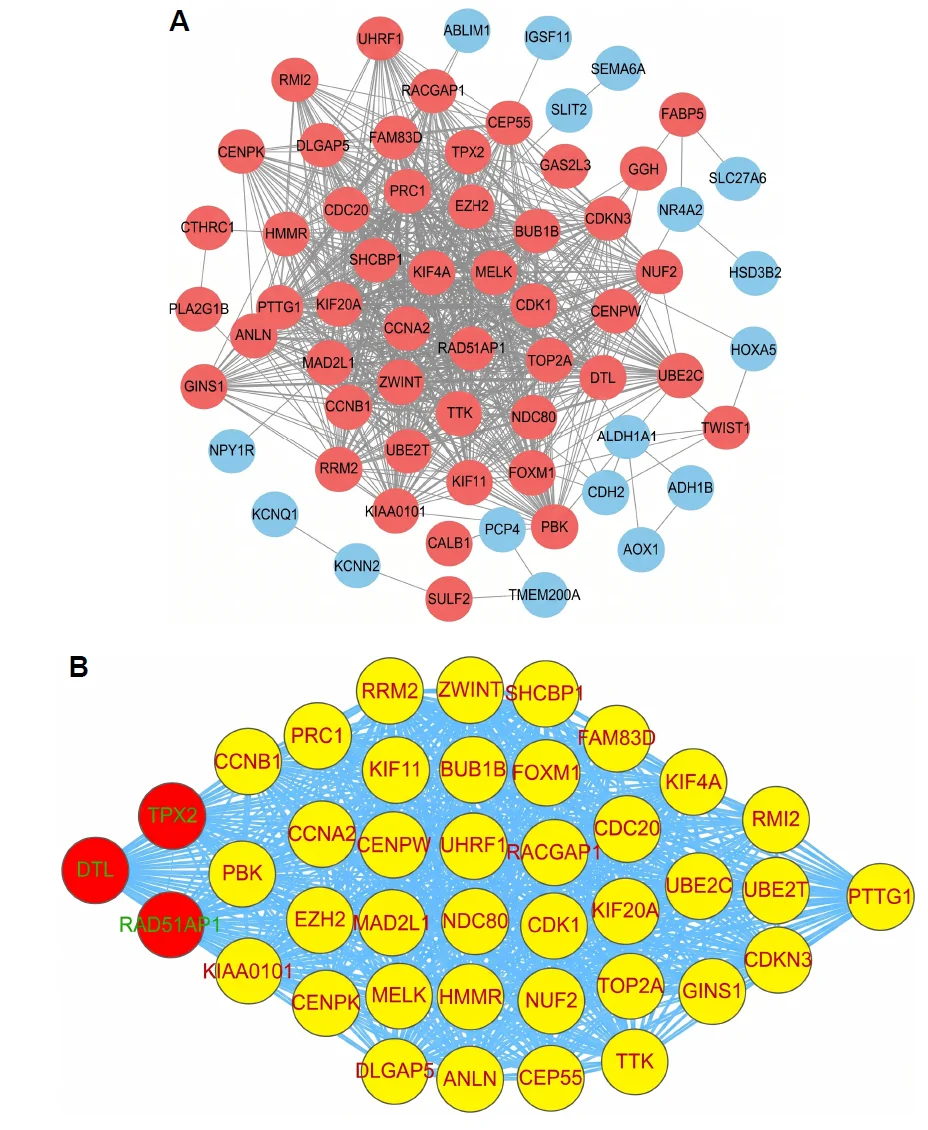
(**A**) PPI network of DEGs. Red nodes represents upregulated genes, while blue nodes represents downregulated genes. (**B**) The most significant functional modules within the PPI network.

**Fig. (3):**
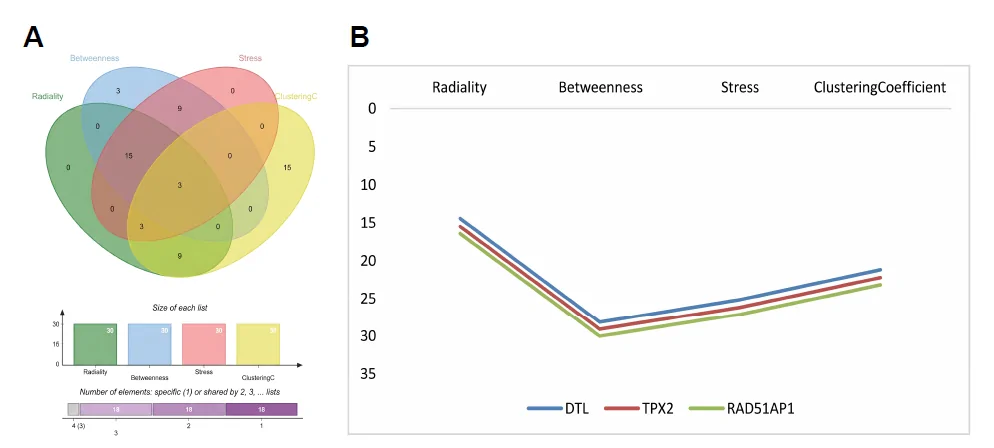
(**A**) Identification of core genes (DTL, TPX2, and RAD51AP1) using four centrality algorithms: degree, betweenness, stress, and clustering coefficient. (**B**) DTL ranks highest among all four centrality algorithms.

**Fig. (4):**
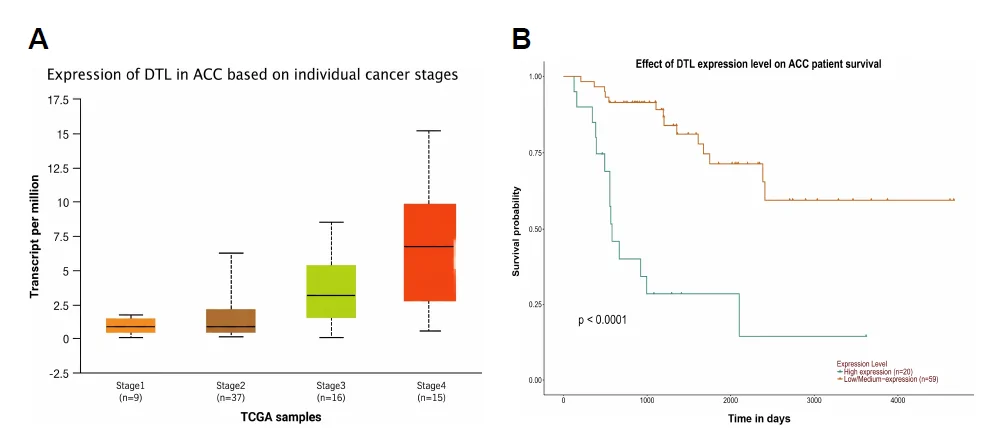
(**A**) Expression levels of the DTL gene across different clinical stages of ACC. (**B**) Kaplan-Meier survival analysis using the UALCAN database, showing overall survival in patients with ACC based on DTL expression levels (red denotes low expression; green denotes high expression).

**Fig. (5):**
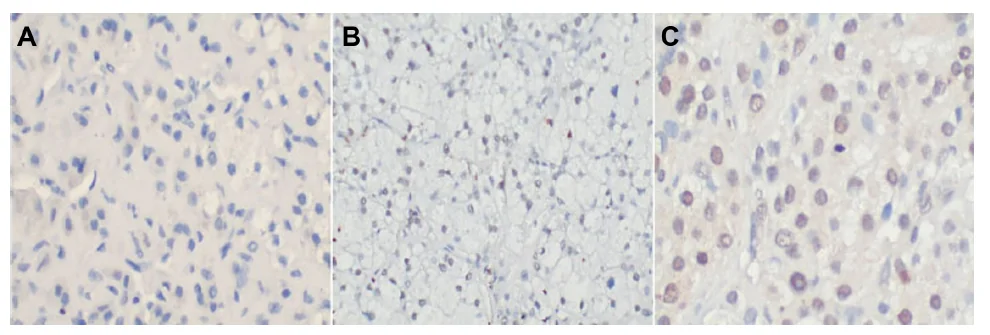
(**A**) DTL is not expressed in normal tissues. (**B**) DTL is expressed in ACA. (**C**) DTL is expressed in ACC.

**Fig. (6):**
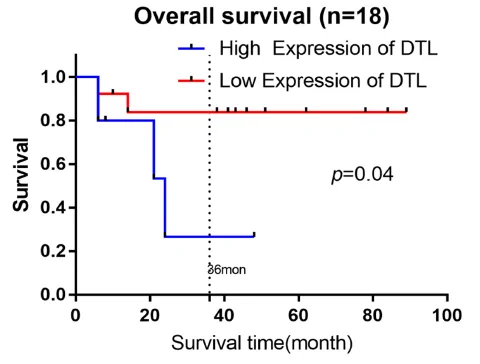
Kaplan-Meier survival curves for patients with ACC stratified by different DTL expression levels. The blue curve represents the high DTL expression group, while the red curve represents the low DTL expression group.

**Table 1:** DTL expression in adrenal tumors and adjacent normal tissues (%).

**Group**	**Cases**	**DTL**				
		**-**	**+**	**++**	**+++**	**Positive Rate (%)**
Carcinoma#*	18	2 (11.11)	12 (66.67)	2 (11.11)	2 (11.11)	88.89
Adenoma^△^	31	19 (61.3)	12 (38.7)	0 (0)	0 (0)	38.7
Adjacent normal adrenal grand	8	8(100.00)	0 (0.00)	0 (0.00)	0 (0.00)	0.00

** Note:** Negative and positive expression of DTL were compared using the Chi-square test, * Carcinoma *vs.* Normal: *P* = 0.000; Fisher’s exact test, # Carcinoma *vs.* Adenoma: χ^2^ = 11.708, *P* = 0.001; Fisher's exact test, ^△^ Adenoma *vs.* Normal: *P* = 0.042.

**Table 2:** Relationship between DTL expression and clinicopathological features in patients with adrenocortical tumors.

**Clinical features**	**N**	**DTL**					
		**-**	**+**	**++**	**+++**	**u**	***P* value**
**Gender**							
Male	19	8	8	2	1	269.50	0.729
Female	30	14	12	3	1		
**Age**							
≥ 42 years old	23	11	8	3	1	294	0.913
< 42 years old	26	11	12	2	1		
**Primary Tumor (pT)**							
<5cm	33	20	12	0	1	107.50	0.000
≥5cm	16	2	8	5	1		
**Infiltration metastasis**							
Yes	7	1	3	2	1	74.50	0.024
No	42	21	17	3	1		
**Clinical stage**							
I,II	9	0	5	3	1	31.5	0.39
III,IV	9	2	4	2	1		

## Data Availability

The data that support the findings of this study are available from the corresponding author upon reasonable request.

## LIST OF ABBREVIATIONS

ACC: Adrenocortical Carcinoma
ACA: Adrenocortical Adenoma
GEO: Gene Expression Omnibus
*DTL*: Denticleless E3 ubiquitin Protein Ligase
